# Image-Guided Stereotactic Body Radiotherapy on Detectable Prostate Bed Recurrence after Prostatectomy in RT-Naïve Patients

**DOI:** 10.3390/life14070870

**Published:** 2024-07-11

**Authors:** Riccardo Santamaria, Mattia Zaffaroni, Maria Giulia Vincini, Lorenzo Colombi, Aurora Gaeta, Federico Mastroleo, Giulia Corrao, Dario Zerini, Riccardo Villa, Giovanni Carlo Mazzola, Sarah Alessi, Stefano Luzzago, Francesco Alessandro Mistretta, Gennaro Musi, Ottavio De Cobelli, Sara Gandini, Lukasz Kuncman, Federica Cattani, Francesco Ceci, Giuseppe Petralia, Giulia Marvaso, Barbara Alicja Jereczek-Fossa

**Affiliations:** 1 Division of Radiation Oncology, IEO European Institute of Oncology IRCCS, 20139 Milan, Italy; riccardo.santamaria@ieo.it (R.S.); mattia.zaffaroni@ieo.it (M.Z.); lorenzo.colombi@ieo.it (L.C.); federico.mastroleo@ieo.it (F.M.); giulia.corrao@ieo.it (G.C.); dario.zerini@ieo.it (D.Z.); riccardo.villa@ieo.it (R.V.); giovannicarlo.mazzola@ieo.it (G.C.M.); giulia.marvaso@ieo.it (G.M.); barbara.jereczek@ieo.it (B.A.J.-F.); 2 Department of Oncology and Hemato-Oncology, University of Milan, 20122 Milan, Italy; stefano.luzzago@ieo.it (S.L.); francescoalessandro.mistretta@ieo.it (F.A.M.); gennaro.musi@ieo.it (G.M.); ottavio.decobelli@ieo.it (O.D.C.); francesco.ceci@ieo.it (F.C.); giuseppe.petralia@ieo.it (G.P.); 3 Department of Experimental Oncology, European Institute of Oncology IRCCS, 20139 Milan, Italy; aurora.gaeta@ieo.it (A.G.); sara.gandini@ieo.it (S.G.); 4 Department of Statistics and Quantitative Methods, University of Milan-Bicocca, 20126 Milan, Italy; 5 Division of Radiology, European Institute of Oncology IRCCS, 20139 Milan, Italy; sarah.alessi@ieo.it; 6 Division of Urology, European Institute of Oncology IRCCS, 20139 Milan, Italy; 7 Department of Radiotherapy, Medical University of Lodz, 90-419 Lodz, Poland; lukasz.kuncman@umed.lodz.pl; 8 Department of External Beam Radiotherapy, Nicolaus Copernicus Multidisciplinary Centre for Oncology and Traumatology, 93-513 Lodz, Poland; 9 Medical Physics Unit, European Institute of Oncology IRCCS, 20139 Milan, Italy; federica.cattani@ieo.it; 10 Division of Nuclear Medicine, European Institute of Oncology IRCCS, 20139 Milan, Italy

**Keywords:** SBRT, macroscopic prostate bed recurrence, mpMRI, PSMA-PET, salvage radiotherapy

## Abstract

Purpose or Objective—The aim of the study is to evaluate the efficacy and safety of SBRT on detectable prostate bed recurrence in RT-naïve prostate cancer patients. Materials and methods: Eighty-six patients who underwent SBRT for macroscopic bed recurrence after prostatectomy were retrospectively included. Patients were treated based on mpMRI or choline/PSMA PET. Results: The median time to biochemical relapse (BCR) after RP was 46 months, with a median PSA at restaging of 1.04 ng/mL. Forty-six patients were staged with mpMRI and choline/PSMA PET, while ten and thirty were treated based on PET and MRI only, respectively. Only one late G ≥ 2 GI toxicity was observed. With a median BCR follow-up of 14 months, twenty-nine patients experienced a BCR with a median PSA at recurrence of 1.66 ng/mL and a median survival free from the event of 40.1 months. The median time to BCR was 17.9 months. Twenty-seven patients had clinical relapse (CR), with a median CR follow-up of 16.27 months and a median time to CR of 23.0 months. Biochemical recurrence-free survival at one and two years was 88% and 66%, respectively, while clinical recurrence-free survival at one and two years was 92% and 82%, respectively. Regarding local relapses, seven were in the field of treatment, while eight of them were outside the field of treatment. Conclusions: Data showed that SBRT targeting only the macroscopic bed recurrence instead of the whole prostate bed is safe and effective. Additional data and longer follow-ups will provide a clearer indication of the appropriate treatment and staging methodology for these patients.

## 1. Introduction

Prostate cancer (PCa) represents the most common malignancy in men worldwide, with the highest prevalence in developed countries [1]. Patients affected by clinically localized PCa with an intermediate- and high-risk disease, according to the National Comprehensive Cancer Network (NCCN)’s prostate guidelines [2], can be considered eligible for several local treatment modalities, including surgery, external-beam radiotherapy or brachytherapy ±, and androgen deprivation therapy (ADT). Surgery by means of radical prostatectomy (RP) represents one of the most widely used treatment options for localized disease and is associated with excellent long-term outcomes [3]. Nonetheless, a large number of patients receiving RP harbor aggressive disease [4,5] and surgery alone might not provide adequate long-term oncological control. A multimodal approach that includes adjuvant or salvage radiotherapy might be considered. Based on the RADICALS-RT, GETUG-AFU-17, and RAVES trials [6,7,8], as well as the ARTISTIC meta-analysis [9], no discernible differences in event-free survival related to a progression of PCa between adjuvant and salvage radiotherapy have been observed. Based on these results and given the potential short- and long-term side effects associated with adjuvant radiotherapy [10,11], salvage and early-salvage approaches represent the preferred treatment choice for PCa patients whose prostate-specific antigen (PSA) levels remain high after RP and for those who develop biochemical recurrence (BCR) [12]. As a matter of fact, BCR rates after surgery are about 30%, with a particularly high risk for patients with positive margins and poorly differentiated disease [13], and their management with curative intent through salvage radiotherapy [14,15,16,17] has appeared to be a highly effective treatment with progression-free survival (PFS) after conventional salvage radiotherapy, without concomitant ADT, of 56% at 5 years [18]. However, in 20–25% of these patients, the recurrence is located outside of the clinical target volume (CTV) [19,20]. The advent of more advanced imaging techniques such as magnetic resonance imaging (mpMRI) and positron emission tomography (PET)-CT with choline or prostate-specific membrane antigen (PSMA) tracers has improved the capacity for effectively detecting the location—whether local, nodal, or distant—of the recurrence. This is of particular importance in helping clinicians to accurately determine the topography of the lesion(s), allowing the delivery of high-dose radiation to eradicate the local disease. Furthermore, recent literature has proposed that the outcome after conventional salvage radiotherapy is potentially worse in patients with positive imaging detecting macroscopic local recurrence at biochemical relapse [21], and the poor outcomes in this subgroup of patients represent an unmet clinical need. In this setting, intensification of local treatment with extremely hypofractionated regimens administered using stereotactic techniques could significantly improve disease control [22,23]. In particular, mpMRI has yielded excellent results in detecting local recurrences and in helping to define target volumes in salvage treatments [24].

In this context, stereotactic body radiotherapy (SBRT) for macroscopic prostate recurrence with image guidance may be used to improve the outcome in these patients, with potential advantages in terms of reduced treatment volumes, normal tissue injury, and lower overall treatment time. Despite being an important clinical scenario, still, no consensus exists regarding dose-escalated radiotherapy for macroscopic recurrence in the prostate bed [25]. The aim of the present study is to evaluate the efficacy and safety of SBRT on detectable prostate bed recurrence in a cohort of RT-naïve patients while also evaluating eventual factors associated with the risk of recurrence.

## 2. Patients and Methods

### 2.1. Inclusion Criteria

Records of men with a diagnosis of PCa who underwent SBRT for macroscopic bed recurrence at the European Institute of Oncology (IEO) IRCCS, Milan, Italy, after radical prostatectomy between December 2014 and June 2022 were retrospectively considered for study inclusion. The inclusion criteria were as follows:

- BCR after previous radical prostatectomy (PSA threshold of 0.2 ng/mL);
- No previous radiotherapy on the prostate bed;
- Isolated macroscopic recurrence in the prostate bed detected by mpMRI or PET (PSMA or choline) after previous surgery;
- No regional or distant recurrence;
- Follow-up ≥ 2 months;
- Age > 18 years;
- Signed written informed consent;
- Any type of prior or concomitant hormone therapy was allowed.

The diagnosis of macroscopic local recurrence as determined by MRI or/and choline or PSMA CT-PET was obtained after the diagnosis of BCR, in accordance with the criteria of the European Association of Urology [26]. Concomitant ADT was allowed. For a recurring lesion, no alternative local therapy was allowed. The analysis only included patients who had a follow-up of at least two months. The ethical committee of the IEO in Milan, Italy, accepted the study as a component of the retrospective research on prostate cancer (notice no. UID 4220). Every patient provided permission for their anonymized data to be used for teaching and research. Adverse occurrences, clinical results, and baseline patient characteristics were gathered and documented.

### 2.2. Radiotherapy Treatment

The gross tumor volume (GTV) corresponded to macroscopic neoplastic tissue in the prostate bed. Assuming that the CTV would be the GTV plus 2 mm, the planning target volume (PTV) consisted of a volumetric expansion of the CTV by 3–5 mm (1–3 mm in the posterior direction). The bladder was excluded from the tumor volume. The following organs at risk were contoured: rectum, bladder, penis, penile bulb, testicles, femoral heads, and bowel.

Patients were treated with SBRT with a total dose of 30–40 Gy in five fractions, corresponding to a BED of 150 Gy and 198.3 Gy (α/β = 1.5), respectively. No simultaneous integrated boost was delivered.

Patients were treated with the VERO system (Vero, BrainLab AG, Feldkirchen, Germany, and Mitsubishi Heavy Industries, Ltd., Tokyo, Japan).

### 2.3. Oncological Outcomes and Follow-Up

Regarding BCR, two different definitions were employed. For patients who received SBRT only, BCR was defined as a PSA level increase of ≥10% when compared to the pre-SBRT value [27]. For patients who received ADT, BCR was defined as a PSA level increase of >0.2 ng/mL [13]. Every three months, patients underwent a clinical evaluation and a PSA blood test. At each visit, toxicities related to the bladder, intestines, and rectal area were noted. Toxicity data were gathered from the clinical records of patients and reported using the Common Terminology Criteria for Adverse Events (CTCAE) score [28].

“In-field” local clinical recurrence was defined when at least a part of the new lesion was part of the previous PTV, while it was defined as “out-field” when in the prostate lodge territory not enclosed in the PTV in the first stereotactic treatment.

Clinical recurrence was defined as oligometastatic when a number of new metastases (bone or lymph node) between 1 and 5 (inclusive) were identified at staging imaging after systemic relapse.

Recurrence was identified as polymetastatic with the detection of >5 metastases at staging imaging after systemic relapse.

### 2.4. Statistics and Data Analysis

Continuous variables were summarized as median and interquartile range (IQR), while categorical variables were presented with absolute and relative frequencies. Survival curves were constructed using the Kaplan–Meier method. The log-rank test was used to compare the survival times between groups. The following oncological outcomes were taken into account for the analysis: BCR-free survival (bRFS), which was defined as the time from the end of RT to BR or last contact at follow-up; and CR-free survival (cRFS), which was defined as the end of RT to CR or last contact at follow-up. Univariate Cox proportional hazard models were performed, and hazard ratios (HRs) with 95% confidence intervals (CIs) were reported.

## 3. Results

### 3.1. Study Population

A total of 86 patients were included in the analysis. The characteristics of the primary tumor are reported in Table 1. The median age at prostatectomy was 65 years. Some of the patients analyzed in our series were also included in the earlier report of Francolini et al. [13]. For the purpose of this study, their follow-up data have been updated.

The characteristics of prostate bed recurrence are shown in Table 2. Among the 86 patients, 76 underwent mpMRI, and 56 underwent PSMA/choline-PET (28 with choline tracer and 28 with PSMA ^68^Ga) as staging imaging. The median time to biochemical relapse (BR) after RP was 46 months (IQR 22–94), with a median PSA at restaging of 1.04 ng/mL (IQR 0.45, 1.93). Forty-six patients (53%) were staged with both mpMRI and choline/PSMA PET, while ten (12%) and thirty (35%) patients were treated based on PET or MRI only, respectively. The median tumor volume (from MRI data available for 63 patients) was 369.07 mm^3^ (IQR 113.04–904.32).

### 3.2. Treatment Characteristics and Toxicity

A summary of the main treatment characteristics and reported toxicities is shown in Table 3. The median age at SBRT treatment was 71.3 years. The majority (91%) of patients underwent a schedule of 35 Gy in 5 fractions (BED = 198.3 Gy). Concomitant hormonal therapy was administered to twelve (14%) of the patients.

Concerning acute toxicities, fifteen patients experienced Grade (G) 1 genitourinary (GU) toxicities, while only one patient reported acute GU G2 toxicity. Nine patients had acute G1 gastrointestinal (GI) toxicities. No G ≥ 3 GU/GI acute toxicities were reported. At a median follow-up of 17.9 months (range 3.9–99.2 months), only one late G ≥ 2 GI toxicity was observed.

### 3.3. Oncological Outcomes

With a median BCR follow-up of 14 months (IQR 9.7–28.5 months), 29 (34%) patients experienced a BCR with a median survival free from the event of 40.1 months (CI 25.2–NA months). The median time to BCR was 17.9 months (range 5.3–69.1 months) and the median PSA at recurrence was 1.66 ng/mL (IQR 0.79, 2.83).

Twenty-seven (26%) patients had CR with a median CR follow-up of 16.27 months (IQR 9.9–28.9 months). The median time to CR was 23.0 months (range 6.0–69.3 months). Four patients were lost at follow-up and were not considered for the Kaplan–Meier analysis. Data on oncological outcomes are reported in Table 4.

**Figure 1:**
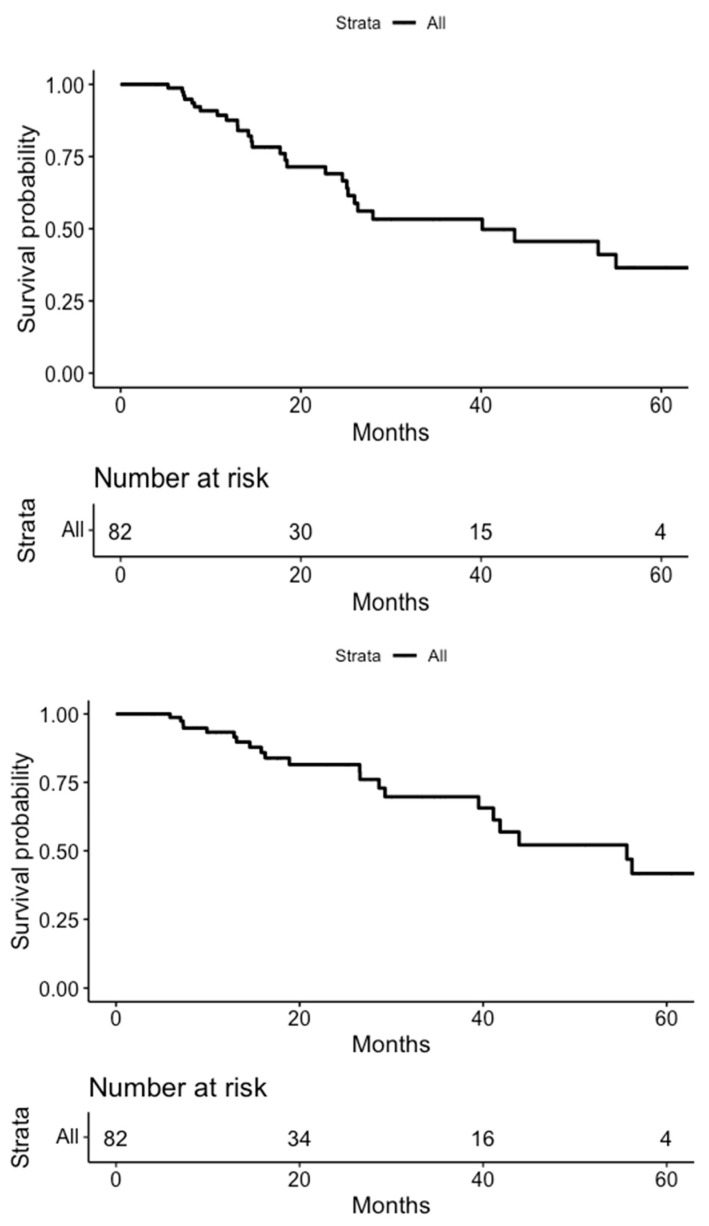
Kaplan–Meier curves for biochemical (**top**) and clinical (**bottom**) recurrence-free survival.

BCR-free survival (bRFS) at one and two years was 88% and 66%, respectively, while CR-free survival (cRFS) at one year was 92% and at two years was 82% (Figure 1). Among the twenty-seven patients who experienced CR, fifteen had a local relapse, nine had an oligometastatic relapse, and three had polymetastatic relapse (>5 lesions). Regarding patients with local relapse (n = 15), seven were in the field of treatment, while eight of them were out-field. Interestingly, all six patients receiving 6 Gy/5 fx developed a local recurrence, and five of them were in-field.

**Figure 2:**
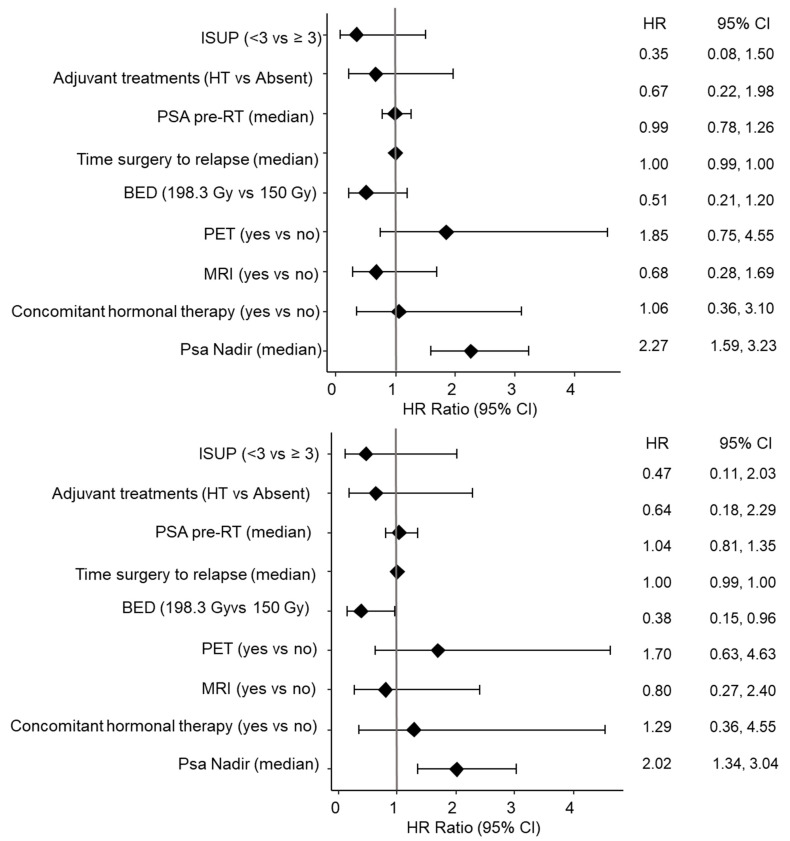
Univariate Cox regression analysis of biochemical (**top**) and clinical (**bottom**) relapse. Abbreviations: HR, hazard ratio; CI, confidence interval; ISUP, International Society of Urological Pathology; HT, hormone therapy; PSA, prostate-specific antigen; BED, biologically effective dose; PET, positron emission tomography; MRI, magnetic resonance imaging.

When investigating clinically relevant features associated with the risk of biochemical and clinical recurrence, only PSA nadir was significantly associated with an increased risk of BCR and CR (Figure 2).

At the last follow-up (data available for 81 patients), 26 (30%) patients were alive with disease (AWD), while 55 (64%) were alive with no evidence of disease (NED). The median PSA at the last FU was 0.20 ng/mL (IQR 0.06–1.01). The PSA trend for every 3 months of follow-up is shown in Figure S1.

### 3.4. Secondary Analyses

**Figure 3:**
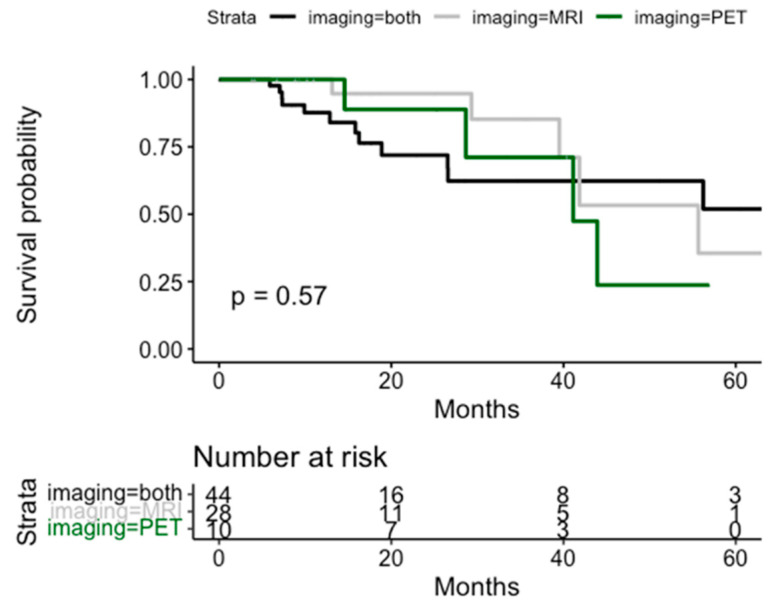
Kaplan–Meier curves for clinical recurrence-free survival according to the type of staging imaging.

**Figure 4:**
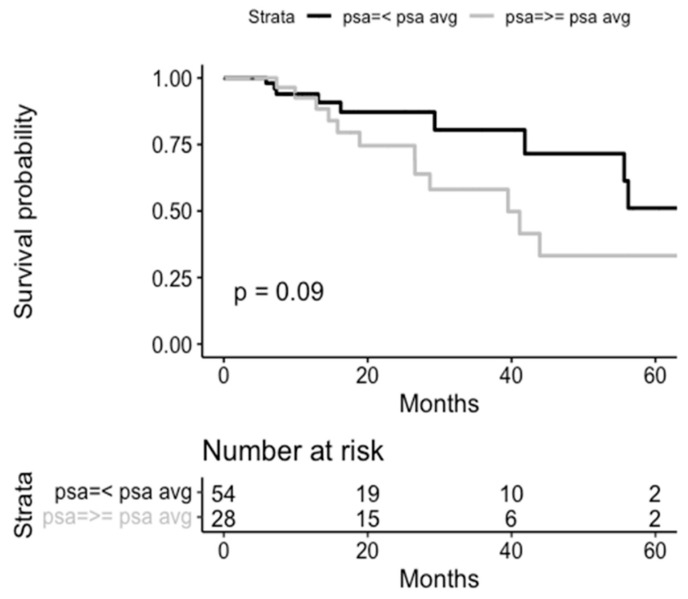
Kaplan–Meier curves for clinical recurrence-free survival according to average PSA at SBRT treatment.

**Figure 5:**
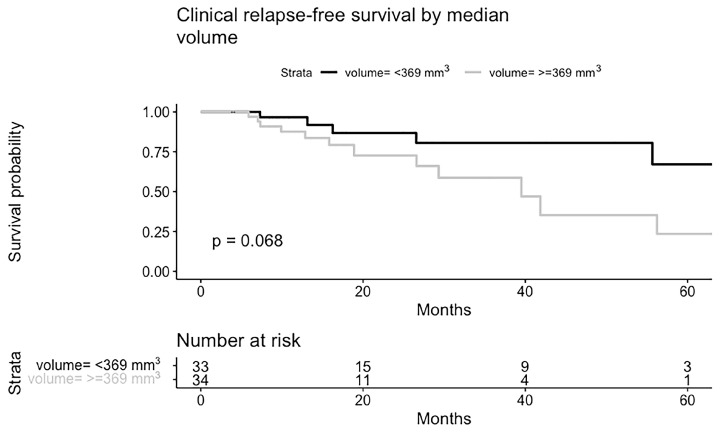
Kaplan–Meier curves for clinical recurrence-free survival according to median tumor volume.

CR-free survival by type of staging for the assessment of isolated relapse in the prostatic bed is shown in Figure 3 and Figure S2. No statistically significant differences were found. No significant differences were found in the CR according to the PSA and tumor volume at SBRT treatment (Figure 4 and Figure 5).

The difference in survival probability when stratifying patients according to BED (198.3 Gy vs. 150 Gy) was statistically significant (*p* = 0.03) in favor of patients who received a BED greater than 198.3 Gy (Supplementary Materials, Figure S3). No further statistically significant differences were found when stratifying patients according to the Gleason score (6–7 vs. 8–9) and adjuvant treatment on the primary (yes vs. no) (Supplementary Materials, Figures S4 and S5).

## 4. Discussion

The present work reports data on a cohort of 86 RT-naïve patients homogeneously treated with image-guided SBRT for macroscopic relapse in the prostate bed. Our data show that targeting macroscopic bed recurrence with SBRT is safe and effective, with negligible toxicities and good rates of oncological control, with the bRFS at one and two years being 88% and 66%, respectively. In our series, tumor control seems slightly lower than that observed in the reports of salvage radiotherapy delivered to the whole prostate bed with or without the pelvic lymph node area. A relatively high PSA level at the SBRT for local recurrence (1.04 ng/mL) can at least partially explain this difference. Indeed, the recent guidelines recommend early salvage radiotherapy at a PSA level of 0.1 ng/mL, since a higher PSA level correlates with lower tumor control [9,26]. Moreover, according to recent evidence from the randomized trials, the addition of ADT to salvage radiotherapy is indicated if the PSA level is above 0.7 ng/mL [29,30]. In our series despite initial PSA of 1.04 ng/mL, only 14% of patients received concomitant ADT. For these two features (high PSA level and lack of ADT in the majority of patients), our results cannot be directly compared to the standard salvage radiotherapy series. Instead, they can be compared to the series of ablative SBRT in patients with any visible recurrence (nodal or local) [27,31]. Notably, when this strategy was compared to traditional salvage radiation in a propensity score-matched analysis, the outcomes [32] did not reveal a statistically significant difference in terms of biochemical relapse-free and progression-free survival. Interestingly, biochemical control was reported to be improved with dose-escalated salvage radiotherapy on the prostate bed when compared with SBRT for macroscopic recurrence (HR = 2.15 [0.63–7.25], *p* = 0.21).

**Figure 6:**
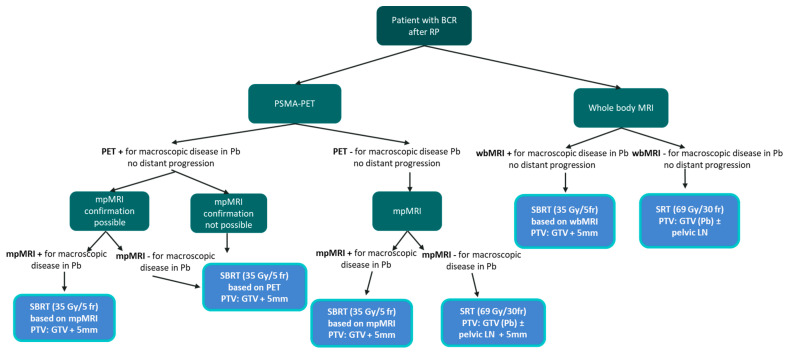
Workflow for patients with macroscopic recurrence in prostate bed after radical prostatectomy.

**Figure 7:**
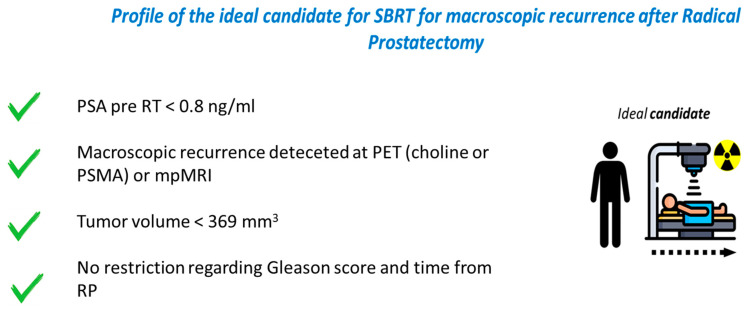
Summary of the main characteristics of the ideal candidate for SBRT for macroscopic recurrence in the prostate bed.

Based on the reported results, we proposed a workflow to be used in everyday clinical practice to treat patients with a macroscopic recurrence in the prostate bed after previous surgery (Figure 6). In addition, our results allowed for the identification of the main characteristics of the ideal patients to be offered SBRT for the macroscopic lesion in the prostate bed (Figure 7). For these particular sets of patients, in case of biochemical recurrence and macroscopic local target, we proposed a personalized treatment with ablative SBRT, by analogy to similar scenarios when next-generation imaging demonstrates oligorecurrence [31].

Salvage radiotherapy for BCR is widely offered as a therapeutic approach and curative treatment for PCa relapse after RP, but the correct approach for salvage radiotherapy is still a matter of debate. In recent years, SBRT to the visible lesion has been considered an attractive alternative to conventional salvage radiotherapy in patients affected by macroscopic prostate bed recurrence after RP [33]. This is of particular importance given the fact that the occurrence of macroscopic recurrence is associated with poor response to standard salvage radiotherapy and the whole prostate bed [19,21]. In addition, the wider implementation of new imaging modalities such as mpMRI and PSMA PET for re-staging after RP allowed for more precise detection of macroscopic evidence of tumor tissue within the prostate bed even at low PSA values. 

Counago et al. [24], in a retrospective analysis of 38 patients with BCR after RP, showed that the combination of both MRI and ^18^FCH PET-CT gives a better local relapse detection rate versus choline PET/CT alone. The inferiority of ^18^FCH-PET compared with ^68^GaPSMA-11-PET was demonstrated for the imaging of recurrent PCa due to the excellent diagnostic accuracy of the latter in this setting, especially at low PSA values [34]. PSMA-PET offers high detection rates and has evolved rapidly to become the gold standard in the staging of biochemically relapsed patients, detecting recurrences in 60–70% of patients with PSA levels < 1 ng/mL [24,35,36].

Meijer et al. [37] showed improved oncological outcomes for patients who received pre-SRT PSMA PET/CT in biochemically recurrent prostate cancer; patients without PSMA PET/CT had a biochemical progression rate of 21% after one year, compared to 8% with pre-SBRT PSMA PET/CT. In a recent study by Tamburo et al., data on 33 patients with recurrent PCa demonstrated that PSMA PET/CT-guided salvage RT can achieve good oncological outcomes with a complete clinical response in 70% of the patients 1 year after the treatment [38]. In addition, Emmet et al. [21] demonstrated the prognostic value of PSMA PET/CT for the evaluation of the treatment response to SBRT in patients with BR. In the same setting, mpMRI imaging with functional sequences allows early detection of local recurrence and may also be a valuable correlative imaging modality for equivocal PET findings [39]. Moreover, the false negative rates of PSMA PET/CT in prostatic ductal adenocarcinoma should not be overlooked, which could result in inadequate clinical staging [40], and the integration of different imaging data could enable an overall improvement in disease detection.

In addition, mpMRI provides a better anatomical delineation of recurrence for the CTV delineation [41], and it allows the delivery of higher radiation doses. In another recent study by Counago et al. [24], pelvic mpMRI was able to detect the location of the recurrence in 33% of patients in a cohort with a median PSA of 0.4 ng/mL. This finding is consistent with the available literature, which reports detection rates for mpMRI ranging from 20 to 40% for patients with low PSA values [42]. In the present study, no significant difference regarding oncological outcomes was found regarding the imaging approach used for staging (PET vs. MRI vs. both). Nevertheless, the underlying potential benefit of combining the advantage of MRI in obtaining morpho-functional information and performing a better GTV delineation and those of PET to detect nodal or distant recurrences should not be overlooked.

Currently, there is widespread support for dose escalation for traditional salvage radiation therapy with the goal of bettering disease control [35,43]; although, there is disagreement about how to appropriately treat macroscopic relapses found in the prostate bed. In actuality, a consensus about the definition of the target volume and the ideal salvage radiotherapy dose has not been adequately established because of differences in methodology and dose constraints [17,26,44]. The prescribed treatment doses in studies similar to the present study ranged between 30 and 35 Gy in five to six fractions [13,45,46,47] with satisfactory results. However, it should be noted that in our study, patients treated with lower BED (i.e., 6–6.5 Gy for five fractions) were more prone to develop a relapse in general and an in-field recurrence in particular.

While dose-escalated conventional salvage radiotherapy was proposed in various retrospective studies reporting BCR-free survival values between 44% and 89% and late G3 toxicities ranging between 2% and 7% [26,43,48,49,50,51,52], currently, SBRT for macroscopic relapse after prostatectomy is not considered a standard approach and is largely restricted to clinical trials [33]. As a consequence, to date, few data are available with regard to long-term outcomes after SBRT to the macroscopic recurrence post-surgery. SBRT has been proposed as an alternative to conventional salvage radiotherapy only in one previous multicentric retrospective series by Francolini et al. [13], where, in a cohort comparable to ours in terms of numbers, an overall biochemical-free survival rate of 72% was reported with an average follow up of 21.2 months and with no G > 2 adverse events registered. With respect to the only study on this setting, the bRFS in our cohort (which included some of the patients included in the study by Francolini et al. [13]) was comparable, with a 20-month bRFS rate of 72% and only one G2 event. It is worth noting that patients included in the study by Francolini et al. [13] presented with a median pre-SBRT PSA level of 2.3 ng/mL higher when compared to our cohort (1.04 ng/mL). Additionally, two prospective phase I studies testing SBRT to prostate bed showed that a dose escalation of up to 35–45 Gy was feasible with a good toxicity profile [45,53]. In particular, a phase I dosage escalation study by Ballas and colleagues [45] aimed to determine the maximum tolerated dose for hypofractionation to the prostate bed. Three different dosage levels were examined by the authors on a cohort of twenty-four patients who had undergone at least six months of follow-up: 54 Gy in 15 fractions, 47 Gy in 10 fractions, and 35.5 Gy in 5 fractions. The findings reported no G ≥ 3 GI or GU toxicity at any dosage. In the 7.1 Gy × 5 fractions cohort, seven out of twelve patients exhibited G2 GI toxicity during treatment, and one patient out of twelve showed an increase in G1 and G2 GU toxicity in the two weeks following RT. Another dose-escalation experiment involving patients with organ-confined, node-negative prostate cancer who experienced biochemical failure following prostatectomy and had a PSA ≤ 2 ng/mL was published by Sampath et al. [53]. The dosage escalation regimen treated their group with 35 Gy, 40 Gy, and 45 Gy in five fractions every other day. Following the inclusion of 26 patients, the median follow-up for the cohorts receiving 35, 40, and 45 Gy was 60, 48, and 33 months, respectively. The findings showed that no acute dose-limiting toxicity events were seen, but 11% and 0% of patients, respectively, had late G ≥ 2 and ≥ 3 GI toxicity, while 38% and 15% of patients reported late grade ≥ 2 and ≥3 GU toxicity. It is noteworthy that there was no increase in late GU toxicity when comparing the 45 Gy group to the 40 Gy cohort, and 42% was the crude rate of full biochemical response. Larger target volumes may have a negative impact on the rate of adverse events in those Phase I trials, making a comparison between these two studies and the current study challenging because they both included all prostate beds within the treatment volume and only included patients affected by biochemical recurrence.

As demonstrated by a multicentric retrospective experience published in 2022, the SPIDER 01 study [54], therapy intensification of some kind seems to be effective for these individuals despite the considerable variability of treatment options offered. The authors of this study gathered information on 363 patients who were treated at 16 centers throughout Europe for biochemical recurrence and macroscopic relapse in the prostate bed, as confirmed by functional MRI. Based on the type of treatment administered, patients treated between January 2000 and December 2019 were split into four groups: no dose escalation, dose escalation on macroscopic recurrence, dose escalation on the prostate bed, and dose escalation on both the prostate bed and macroscopic recurrence. Notably, the results indicated that all groups with any dose escalation > 72 Gy had a five-year progression-free survival benefit (72.8% vs. 60.3%, *p* = 0.03). This suggests that when macroscopic relapse is detected inside the prostate bed, functional imaging integration in the salvage treatment approach is effective, and that dose escalation had a significant effect on progression-free survival.

Thus, treatment intensification for patients with macroscopic prostate bed recurrence appears justified, but the correct approach is still to be defined. Possibly the results of some of the ongoing trials will shed light on the matter.

The ongoing STARR trial enrolls patients treated with RP for localized prostate cancer and affected by macroscopic recurrence within the prostate bed detected by choline- or PSMA-PET and confirmed by mpMRI. The early results of the trial on 25 patients showed encouraging results with regard to biochemical control, with biochemical response detected at 3 months in 84.3% of the patients [55], but a larger cohort and a longer follow-up will provide clearer indications. The currently ongoing POPART trial (NCT04831970), a multicentric prospective observational trial, enrolls patients with biochemical and/or clinical relapse following RP with the main aim of investigating the feasibility of ultra-hypofractionated radiotherapy to the prostate bed in this set of patients. The preliminary reports on toxicity and quality of life are promising, without an increase in short-term toxicity or a significant decline in quality of life, and warrant long-term data to confirm the feasibility of this treatment strategy. Finally, to compare the toxicity rates of the two approaches, a further ongoing prospective trial (SHORTER, NCT04422132) is currently enrolling patients who have been randomly assigned to receive either SBRT (32.5 Gy in 5 fractions) or moderate hypofractionation (55 Gy in 20 fractions) to the prostate bed ± pelvic nodes. The present study is not exempt from limitations. First and foremost, the retrospective nature of the analysis and the relatively limited follow-up duration. Nevertheless, it is the second-largest study on this setting with a homogeneous and well-selected real-world study cohort.

## 5. Conclusions

To conclude, considering the favorable therapeutic ratio of this approach reported in the literature [13,32] and in the present study, SBRT for the visible lesion should be considered an attractive alternative to conventional and dose-escalated salvage radiotherapy for the prostate bed in patients affected by macroscopic prostate bed recurrence after RP [33]. To ulteriorly improve the oncological outcomes, given the encouraging toxicity results for dose-escalation on the entire prostate bed, a future perspective might be to treat the entire lodge with SBRT, with a boost on the macroscopic lesions. More robust data from randomized controlled trials will hopefully provide clearer indications regarding the most appropriate techniques, dose constraints, strategies for target volume definition, and patient selection.

## Figures and Tables

**Table 1:** Summary of patients’ characteristics.

Primary tumor	**n = 86**		**Median (IQR)**
	**age at diagnosis**		65 (61, 69)
	**PSA post OP (ng/mL)**		0.03 (0.01, 0.08)
	missing		2
			**n (%)**
	**pT**	2	40 (47%)
		3	43 (49%)
		4	1 (1.2%)
		x	2 (2.3%)
	**pN**	0	72 (84%)
		1	10 (12%)
		x	4 (4.7%)
	**cM**	0	83 (97%)
		X	3 (3.5%)
	**R**	R0	60 (70%)
		R1	24 (28%)
		X	2 (2.3%)
	**ISUP**	1 = 3 + 3	11 (13%)
		2 = 3 + 4	27 (31%)
		3 = 4 + 3	29 (34%)
		4 = 4 + 4	7 (8.1%)
		5 = 4 + 5 or more	10 (11%+)
		
		X	2 (2.3%)
	**PN**	Absent	64 (74%)
		Present	20 (23%)
		X	2 (2.3%)
	**Post-op ADT (HT)**	No	72 (84%)
		HT	14 (16%)

**Table 2:** Summary of patients’ characteristics at prostate bed relapse.

Bed recurrence	**n = 86**		**Median (IQR)**
	**Time from surgery to recurrence (months)**		46 (22, 94)
	**Initial PSA (ng/mL)**		1.04 (0.45, 1.93)
			n (%)
	**Imaging**	Both MRI and PET	46 (53%)
		MRI only	30 (35%)
		PET only	10 (12%)
	**PET**	No	30 (35%)
		Yes	56 (65%)
	**Tracer**	Choline	28 (50%)
		PSMA	28 (50%)
	**MRI**	No	10 (12%)
		Yes	76 (88%)

**Table 3:** Main treatment characteristics and reported toxicities.

**n = 86**		**Median (IQR)**
**Age at treatment RT**		71.3 (67.2, 74.2)
		**n (%)**
**Schedule**	BED 150 (6 Gy × 5 fr)	4 (4.7%)
	BED 173.3 (6.5 Gy × 5 fr)	4 (4.7%)
	BED 198.3 (7 Gy × 5 fr)	78 (91%)
**Concomitant hormonal therapy**	Yes	12 (14%)
	No	74 (86%)
**Hormonal therapy type**	BICALUTAMIDE 150 mg	2 (16%)
	LHRH analog	10 (84%)
		**n (%)**
**Acute GU toxicity**	0	70 (81%)
	1	15 (17%)
	2	1 (1.2%)
**Acute GI toxicity**	0	76 (88%)
	1	9 (10%)
**Late GU toxicity**	0	86 (100%)
**Late GI toxicity**	0	77 (89%)
	1	8 (9.5%)
	2	1 (1.2%)

**Table 4:** Data on oncological outcomes.

	**n = 29**		**Median (IQR)**
Biochemical relapse n = 29 (34%)	**PSA at recurrence**		1.66 (0.79, 2.83)
	**n = 27**		**n (%)**
Clinical relapse n = 27 (26%)	Recurrence Type	local	15 (54%)
		IN FIELD	7 (50%)
		OUTFIELD	8 (50%)
		oligometastatic	9 (35%)
		Polymetastatic (>5)	3 (12%)
	**Re-staging exam**	Choline-PET	5 (19%)
		PSMA-PET	12 (46%)
		Pelvic mpRMI	10 (36%)
		
	**Treatment at CR**	ADT	6 (24%)
		ADT + RT	4 (16%)
		RT	15 (56%)
		Follow-up only	1 (4.0%)
	**n = 86**		**n (%)**
Last FU (n = 86)	AWD		26 (30%)
	NED		55 (64%)
	Missing		5 (5.8%)
			**Median (IQR)**
	**Last PSA (ng/mL)**		0.20 (0.06, 1.01)
	**PSA nadir (ng/mL)**		0.16 (0.07, 0.49)
	**Missing**		16

## Data Availability

The data presented in this study are available on request from the corresponding author.

## Supplementary Materials

The following supporting information can be downloaded at: https://www.mdpi.com/article/10.3390/life14070870/s1, Figure S1: Summary of PSA evolution from baseline to 18 months; Figure S2: Kaplan-Meier curves for clinical recurrence-free survival according to type of staging imaging. (a) PET yes vs. no; (b) MRI yes vs. no; Figure S3. Kaplan-Meier curves for clinical recurrence-free survival according to BED (198.3 Gy vs. 150 Gy); Figure S4. Kaplan-Meier curves for clinical recurrence-free survival according to GS; Figure S5. Kaplan-Meier curves for clinical recurrence-free survival according to concomitant ADT administration (ADT yes vs. no).

## List of Abbreviations

ADT	Androgen Deprivation Therapy
AWD	Alive with disease
BCR	Biochemical Recurrence
BED	Biologically Effective Dose
bRFS	Biochemical Relapse-free Survival
cRFS	Clinical Relapse-free Survival
CR	Clinical Recurrence
CT	Computed Tomography
CTV	clinical target volume
G	Grade
GI	Gastrointestinal
GU	Genitourinary
HR	Hazard Ratio
IQR	Interquartile range
mp	Multi-parametric
MRI	Magnetic Resonance Imaging
NCCN	National Comprehensive Cancer Network
NED	Not evidence of disease
OS	Overall Survival
PCa	Prostate Cancer
PET	Positron Emission Tomography
PFS	Progression-free Survival
PSMA	Prostate-specific Membrane Antigen
RP	radical prostatectomy
SBRT	Stereotactic Body Radiation Therapy

## References

[1] Sung H., Ferlay J., Siegel R.L., Laversanne M., Soerjomataram I., Jemal A., Bray F. (2021). Global Cancer Statistics 2020: GLOBOCAN Estimates of Incidence and Mortality Worldwide for 36 Cancers in 185 Countries. CA Cancer J. Clin..

[2] Schaeffer E., An Y., Barocas D. (2022). NCCN Guidelines Version 1.2023 Prostate Cancer. https://www.nccn.org/home/.

[3] Hamdy F.C., Donovan J.L., Lane J.A., Mason M., Metcalfe C., Holding P., Davis M., Peters T.J., Turner E.L., Martin R.M. (2016). 10-Year Outcomes after Monitoring, Surgery, or Radiotherapy for Localized Prostate Cancer. N. Engl. J. Med..

[4] Sineshaw H.M., Gray P.J., Efstathiou J.A., Jemal A. (2015). Declining Use of Radiotherapy for Adverse Features after Radical Prostatectomy: Results from the National Cancer Data Base. Eur. Urol..

[5] Leyh-Bannurah S.-R., Gazdovich S., Budäus L., Zaffuto E., Dell’Oglio P., Briganti A., Abdollah F., Montorsi F., Schiffmann J., Menon M. (2017). Population-Based External Validation of the Updated 2012 Partin Tables in Contemporary North American Prostate Cancer Patients. Prostate.

[6] Parker C.C., Clarke N.W., Cook A.D., Kynaston H.G., Petersen P.M., Catton C., Cross W., Logue J., Parulekar W., Payne H. (2020). Timing of radiotherapy after radical prostatectomy (RADICALS-RT): A randomised, controlled phase 3 trial. Lancet.

[7] Sargos P., Chabaud S., Latorzeff I., Magné N., Benyoucef A., Supiot S., Pasquier D., Abdiche M.S., Gilliot O., Graff-Cailleaud P. (2020). Adjuvant radiotherapy versus early salvage radiotherapy plus short-term androgen deprivation therapy in men with localised prostate cancer after radical prostatectomy (GETUG-AFU 17): A randomised, phase 3 trial. Lancet Oncol..

[8] Pearse M., Fraser-Browne C., Davis I.D., Duchesne G.M., Fisher R., Frydenberg M., Haworth A., Jose C., Joseph D.J., Lim T.S. (2014). A Phase III trial to investigate the timing of radiotherapy for prostate cancer with high-risk features: Background and rationale of the Radiotherapy—Adjuvant Versus Early Salvage (RAVES) trial. BJU Int..

[9] Vale C.L., Fisher D., Kneebone A., Parker C., Pearse M., Richaud P., Sargos P., Sydes M.R., Brawley C., Brihoum M. (2020). Adjuvant or early salvage radiotherapy for the treatment of localised and locally advanced prostate cancer: A prospectively planned systematic review and meta-analysis of aggregate data. Lancet.

[10] van Stam M.-A., Aaronson N.K., Pos F.J., Bosch J.L.H.R., Kieffer J.M., Tillier C.N., van der Poel H.G. (2016). The Effect of Salvage Radiotherapy and its Timing on the Health-related Quality of Life of Prostate Cancer Patients. Eur. Urol..

[11] Suardi N., Gallina A., Lista G., Gandaglia G., Abdollah F., Capitanio U., Dell’Oglio P., Nini A., Salonia A., Montorsi F. (2014). Impact of adjuvant radiation therapy on urinary continence recovery after radical prostatectomy. Eur. Urol..

[12] Fossati N., Karnes R.J., Boorjian S.A., Moschini M., Morlacco A., Bossi A., Seisen T., Cozzarini C., Fiorino C., Noris Chiorda B. (2017). Long-term Impact of Adjuvant Versus Early Salvage Radiation Therapy in pT3N0 Prostate Cancer Patients Treated with Radical Prostatectomy: Results from a Multi-institutional Series. Eur. Urol..

[13] Francolini G., Jereczek-Fossa B.A., Di Cataldo V., Simontacchi G., Marvaso G., Zerella M.A., Gentile P., Bianciardi F., Allegretta S., Detti B. (2020). Stereotactic radiotherapy for prostate bed recurrence after prostatectomy, a multicentric series. BJU Int..

[14] Thompson I.M., Valicenti R.K., Albertsen P., Davis B.J., Goldenberg S.L., Hahn C., Klein E., Michalski J., Roach M., Sartor O. (2013). Adjuvant and salvage radiotherapy after prostatectomy: AUA/ASTRO Guideline. J. Urol..

[15] Poortmans P., Bossi A., Vandeputte K., Bosset M., Miralbell R., Maingon P., Boehmer D., Budiharto T., Symon Z., van den Bergh A.C.M. (2007). Guidelines for target volume definition in post-operative radiotherapy for prostate cancer, on behalf of the EORTC Radiation Oncology Group. Radiother. Oncol..

[16] Lawton C.A.F., Michalski J., El-Naqa I., Buyyounouski M.K., Lee W.R., Menard C., O’Meara E., Rosenthal S.A., Ritter M., Seider M. (2009). RTOG GU Radiation oncology specialists reach consensus on pelvic lymph node volumes for high-risk prostate cancer. Int. J. Radiat. Oncol. Biol. Phys..

[17] Dal Pra A., Dirix P., Khoo V., Carrie C., Cozzarini C., Fonteyne V., Ghadjar P., Gomez-Iturriaga A., Panebianco V., Zapatero A. (2023). ESTRO ACROP guideline on prostate bed delineation for postoperative radiotherapy in prostate cancer. Clin. Transl. Radiat. Oncol..

[18] Carrie C., Hasbini A., de Laroche G., Richaud P., Guerif S., Latorzeff I., Supiot S., Bosset M., Lagrange J.-L., Beckendorf V. (2016). Salvage radiotherapy with or without short-term hormone therapy for rising prostate-specific antigen concentration after radical prostatectomy (GETUG-AFU 16): A randomised, multicentre, open-label phase 3 trial. Lancet Oncol..

[19] Dirix P., van Walle L., Deckers F., Van Mieghem F., Buelens G., Meijnders P., Huget P., Van Laere S. (2017). Proposal for magnetic resonance imaging-guided salvage radiotherapy for prostate cancer. Acta Oncol..

[20] Calais J., Czernin J., Cao M., Kishan A.U., Hegde J.V., Shaverdian N., Sandler K., Chu F.-I., King C.R., Steinberg M.L. (2018). 68Ga-PSMA-11 PET/CT Mapping of Prostate Cancer Biochemical Recurrence after Radical Prostatectomy in 270 Patients with a PSA Level of Less than 1.0 ng/mL: Impact on Salvage Radiotherapy Planning. J. Nucl. Med..

[21] Emmett L., van Leeuwen P.J., Nandurkar R., Scheltema M.J., Cusick T., Hruby G., Kneebone A., Eade T., Fogarty G., Jagavkar R. (2017). Treatment Outcomes from 68Ga-PSMA PET/CT-Informed Salvage Radiation Treatment in Men with Rising PSA after Radical Prostatectomy: Prognostic Value of a Negative PSMA PET. J. Nucl. Med..

[22] Ohri N., Dicker A.P., Trabulsi E.J., Showalter T.N. (2012). Can early implementation of salvage radiotherapy for prostate cancer improve the therapeutic ratio? A systematic review and regression meta-analysis with radiobiological modelling. Eur. J. Cancer.

[23] King C.R. (2016). The dose-response of salvage radiotherapy following radical prostatectomy: A systematic review and meta-analysis. Radiother. Oncol..

[24] Couñago F., Díaz Gavela A.A., Sancho G., Ortiz I., Marcos F.J., Recio M., Fernández J., Cano R., Jiménez M., Thuissard I.J. (2019). Multiparametric magnetic resonance imaging-guided salvage radiotherapy in prostate cancer. Rep. Pract. Oncol. Radiother..

[25] Dal Pra A., Panje C., Zilli T., Arnold W., Brouwer K., Garcia H., Glatzer M., Gomez S., Herrera F., Kaouthar K. (2018). Salvage radiotherapy for macroscopic local recurrences after radical prostatectomy: A national survey on patterns of practice. Strahlenther. Onkol..

[26] Mottet N., van den Bergh R.C.N., Briers E., Van den Broeck T., Cumberbatch M.G., De Santis M., Fanti S., Fossati N., Gandaglia G., Gillessen S. (2021). EAU-EANM-ESTRO-ESUR-SIOG Guidelines on Prostate Cancer-2020 Update. Part 1: Screening, Diagnosis, and Local Treatment with Curative Intent. Eur. Urol..

[27] Jereczek-Fossa B.A., Beltramo G., Fariselli L., Fodor C., Santoro L., Vavassori A., Zerini D., Gherardi F., Ascione C., Bossi-Zanetti I. (2012). Robotic image-guided stereotactic radiotherapy, for isolated recurrent primary, lymph node or metastatic prostate cancer. Int. J. Radiat. Oncol. Biol. Phys..

[28] Common Terminology Criteria for Adverse Events (CTCAE) Common Terminology Criteria for Adverse Events (CTCAE) v40:196. https://evs.nci.nih.gov/ftp1/CTCAE/CTCAE_4.03/CTCAE_4.03_2010-06-14_QuickReference_8.5x11.pdf.

[29] Pollack A., Karrison T.G., Balogh A.G., Gomella L.G., Low D.A., Bruner D.W., Wefel J.S., Martin A.-G., Michalski J.M., Angyalfi S.J. (2022). The addition of androgen deprivation therapy and pelvic lymph node treatment to prostate bed salvage radiotherapy (NRG Oncology/RTOG 0534 SPPORT): An international, multicentre, randomised phase 3 trial. Lancet.

[30] Burdett S., Fisher D., Parker C.C., Sydes M.R., Pommier P., Sargos P., Spratt D.E., Kishan A.U., Brihoum M., Catton C. (2022). LBA64 Duration of androgen suppression with post-operative radiotherapy (DADSPORT): A collaborative meta-analysis of aggregate data. Ann. Oncol..

[31] Marvaso G., Volpe S., Pepa M., Augugliaro M., Corrao G., Biffi A., Zaffaroni M., Bergamaschi L., La Fauci F.M., Mistretta F.A. (2021). Oligorecurrent Prostate Cancer and Stereotactic Body Radiotherapy: Where Are We Now? A Systematic Review and Meta-analysis of Prospective Studies. Eur. Urol. Open Sci..

[32] Francolini G., Jereczek-Fossa B.A., Di Cataldo V., Simontacchi G., Marvaso G., Gandini S., Corso F., Ciccone L.P., Zerella M.A., Gentile P. (2022). Stereotactic or conventional radiotherapy for macroscopic prostate bed recurrence: A propensity score analysis. Radiol. Med..

[33] Schröder C., Tang H., Windisch P., Zwahlen D.R., Buchali A., Vu E., Bostel T., Sprave T., Zilli T., Murthy V. (2022). Stereotactic Radiotherapy after Radical Prostatectomy in Patients with Prostate Cancer in the Adjuvant or Salvage Setting: A Systematic Review. Cancers.

[34] Hofman M.S., Lawrentschuk N., Francis R.J., Tang C., Vela I., Thomas P., Rutherford N., Martin J.M., Frydenberg M., Shakher R. (2020). Prostate-specific membrane antigen PET-CT in patients with high-risk prostate cancer before curative-intent surgery or radiotherapy (proPSMA): A prospective, randomised, multicentre study. Lancet.

[35] Tamihardja J., Zehner L., Hartrampf P.E., Cirsi S., Wegener S., Buck A.K., Flentje M., Polat B. (2022). Dose-Escalated Salvage Radiotherapy for Macroscopic Local Recurrence of Prostate Cancer in the Prostate-Specific Membrane Antigen Positron Emission Tomography Era. Cancers.

[36] Pepe P., Pepe L., Cosentino S., Ippolito M., Pennisi M., Fraggetta F. (2022). Detection Rate of 68Ga-PSMA PET/CT vs. mpMRI Targeted Biopsy for Clinically Significant Prostate Cancer. Anticancer Res..

[37] Meijer D., Eppinga W.S.C., Mohede R.M., Vanneste B.G.L., Meijnen P., Meijer O.W.M., Daniels L.A., van den Bergh R.C.N., Lont A.P., Ettema R.H. (2022). Prostate-specific Membrane Antigen Positron Emission Tomography/Computed Tomography Is Associated with Improved Oncological Outcome in Men Treated with Salvage Radiation Therapy for Biochemically Recurrent Prostate Cancer. Eur. Urol. Oncol..

[38] Tamburo M., Buffettino E., Pepe P., Marletta G., Pepe L., Cosentino S., Ippolito M., Pennisi M., Marletta F. (2024). Salvage Radi-otherapy PSMA PET/CT-guided in Men with PSA Recurrence. Anticancer Res..

[39] Kitajima K., Murphy R.C., Nathan M.A., Froemming A.T., Hagen C.E., Takahashi N., Kawashima A. (2014). Detection of recurrent prostate cancer after radical prostatectomy: Comparison of 11C-choline PET/CT with pelvic multiparametric MR imaging with endorectal coil. J. Nucl. Med..

[40] Pepe P., Pepe L., Curduman M., Pennisi M., Fraggetta F. (2024). Ductal prostate cancer staging: Role of PSMA PET/CT. Arch Ital. Urol. Androl..

[41] Latorzeff I., Sargos P., Loos G., Supiot S., Guerif S., Carrie C. (2017). Delineation of the Prostate Bed: The “Invisible Target” Is Still an Issue?. Front. Oncol..

[42] Couñago F., Sancho G., Catalá V., Hernández D., Recio M., Montemuiño S., Hernández J.A., Maldonado A., Del Cerro E. (2017). Magnetic resonance imaging for prostate cancer before radical and salvage radiotherapy: What radiation oncologists need to know. World J. Clin. Oncol..

[43] Shelan M., Odermatt S., Bojaxhiu B., Nguyen D.P., Thalmann G.N., Aebersold D.M., Dal Pra A. (2019). Disease Control with Delayed Salvage Radiotherapy for Macroscopic Local Recurrence Following Radical Prostatectomy. Front. Oncol..

[44] Parker C., Castro E., Fizazi K., Heidenreich A., Ost P., Procopio G., Tombal B., Gillessen S., ESMO Guidelines Committee (2020). Prostate cancer: ESMO Clinical Practice Guidelines for diagnosis, treatment and follow-up. Ann. Oncol..

[45] Ballas L.K., Luo C., Chung E., Kishan A.U., Shuryak I., Quinn D.I., Dorff T., Jhimlee S., Chiu R., Abreu A. (2019). Phase 1 Trial of SBRT to the Prostate Fossa after Prostatectomy. Int. J. Radiat. Oncol. Biol. Phys..

[46] Arcangeli S., Gambardella P., Agolli L., Monaco A., Dognini J., Regine G., Donato V. (2015). Stereotactic body radiation therapy salvage reirradiation of radiorecurrent prostatic carcinoma relapsed in the prostatic bed. Tumori.

[47] Jereczek-Fossa B.A., Rojas D.P., Zerini D., Fodor C., Viola A., Fanetti G., Volpe S., Luraschi R., Bazani A., Rondi E. (2019). Reirradiation for isolated local recurrence of prostate cancer: Mono-institutional series of 64 patients treated with salvage stereotactic body radiotherapy (SBRT). Br. J. Radiol..

[48] Bruni A., Ingrosso G., Trippa F., Di Staso M., Lanfranchi B., Rubino L., Parente S., Frassinelli L., Maranzano E., Santoni R. (2019). Macroscopic locoregional relapse from prostate cancer: Which role for salvage radiotherapy?. Clin. Transl. Oncol..

[49] Zaine H., Vandendorpe B., Bataille B., Lacornerie T., Wallet J., Mirabel X., Lartigau E., Pasquier D. (2021). Salvage Radiotherapy for Macroscopic Local Recurrence Following Radical Prostatectomy. Front. Oncol..

[50] Schmidt-Hegemann N.-S., Stief C., Kim T.-H., Eze C., Kirste S., Strouthos I., Li M., Schultze-Seemann W., Ilhan H., Fendler W.P. (2019). Outcome after PSMA PET/CT based salvage radiotherapy in patients with biochemical recurrence after radical prostatectomy: A bi-institutional retrospective analysis. J. Nucl. Med..

[51] Zilli T., Jorcano S., Peguret N., Caparrotti F., Hidalgo A., Khan H.G., Vees H., Miralbell R. (2017). Results of Dose-adapted Salvage Radiotherapy after Radical Prostatectomy Based on an Endorectal MRI Target Definition Model. Am. J. Clin. Oncol..

[52] Lee S.U., Cho K.H., Kim J.H., Kim Y.S., Nam T.-K., Kim J.-S., Cho J., Choi S.H., Shim S.J., Kim J.H. (2021). Clinical Outcome of Salvage Radiotherapy for Locoregional Clinical Recurrence after Radical Prostatectomy. Technol. Cancer Res. Treat..

[53] Sampath S., Frankel P., Vecchio B.D., Ruel N., Yuh B., Liu A., Tsai T., Wong J. (2020). Stereotactic Body Radiation Therapy to the Prostate Bed: Results of a Phase 1 Dose-Escalation Trial. Int. J. Radiat. Oncol. Biol. Phys..

[54] Benziane-Ouaritini N., Zilli T., Ingrosso G., di Staso M., Trippa F., Francolini G., Meyer E., Achard V.M., Schick U., Cosset J.M. (2022). Salvage Radiotherapy Guided by Functional Imaging for Macroscopic Local Recurrence Following Radical Prostatectomy: A Multicentric Retrospective Study. Int. J. Radiat. Oncol. Biol. Phys..

[55] Francolini G., Garlatti P., Cataldo V.D., Detti B., Loi M., Greto D., Simontacchi G., Morelli I., Burchini L., Allegra A.G. (2023). Three Months’ PSA and Toxicity from a Prospective Trial Investigating STereotactic sAlvage Radiotherapy for Macroscopic Prostate Bed Recurrence after Prostatectomy—STARR (NCT05455736). Cancers.

