# Supplementary material for: Image-Guided Stereotactic Body Radiotherapy on Detectable Prostate Bed Recurrence after Prostatectomy in RT-Naïve Patients
Source: Life (Basel). 2024 Jul 11;14(7):870. doi: 10.3390/life14070870 (PMC11277978; doi:10.3390/life14070870)

Figure S1. Summary of PSA evolution from baseline to 18 months

PSA (Ng/ml)	**bl**	**3 m**	**6 m**	**9 m**	**12 m**	**15 m**	**18 m**
Median	0.80	0.50	0.28	0.30	0.29	0.29	0.17
IQR	(0.40 – 1.72)	(0.21 - 0.94)	(0.16 – 0.80)	(0.12 – 0.70)	(0.11 – 0.70)	(0.12 – 0.59)	(0.05 – 0.58)

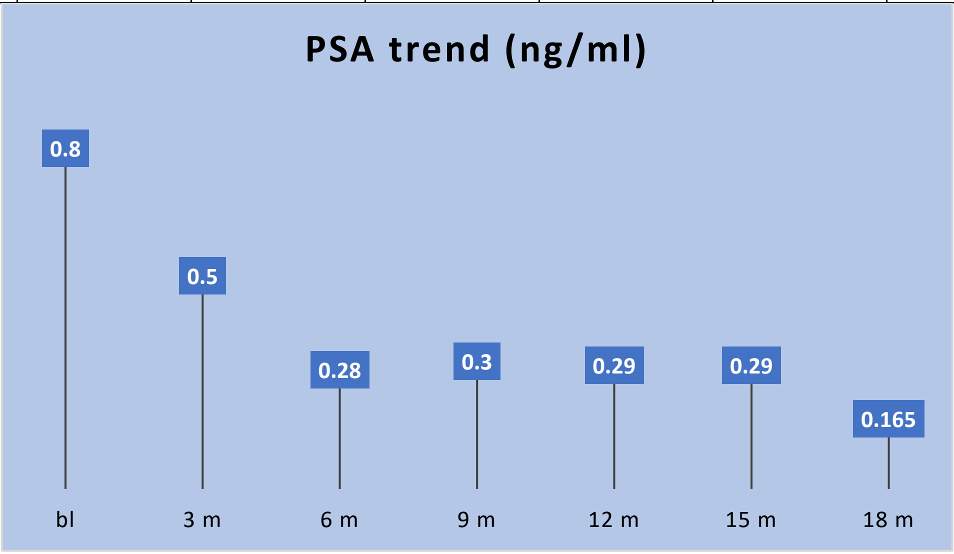

Figure S2. Kaplan-Meier curves for clinical recurrence-free survival according to type of staging imaging. A: PET yes vs no; B: MRI yes vs no;

a.

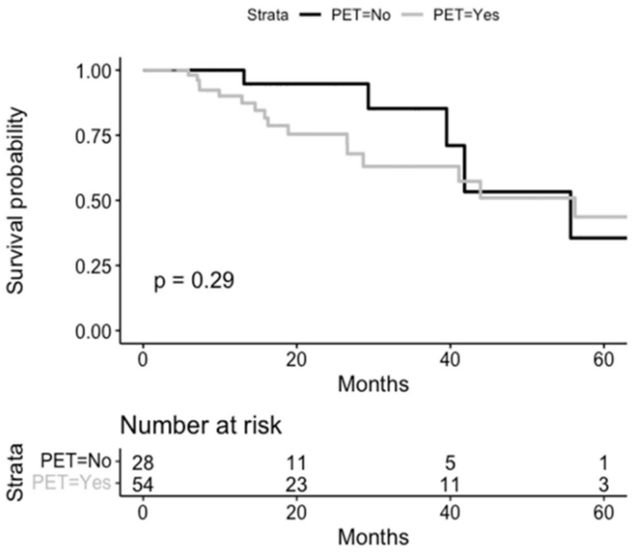

b.

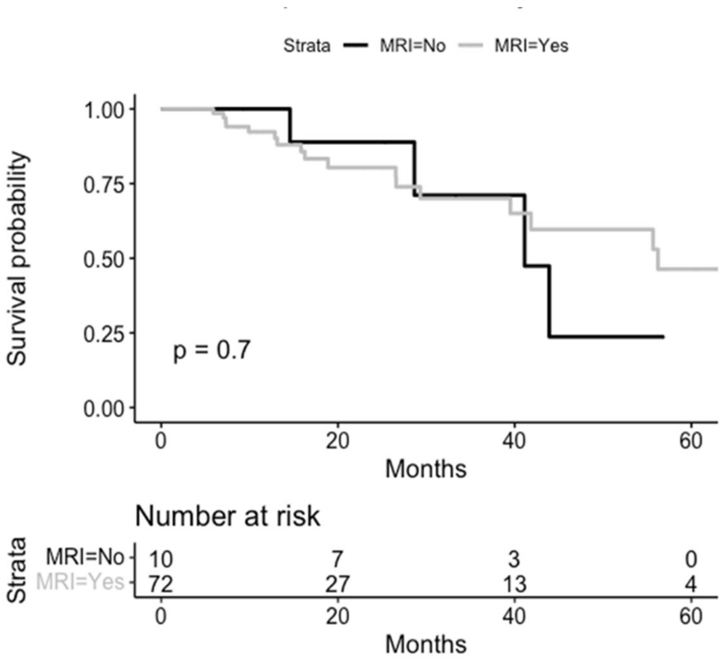

Figure S3. Kaplan-Meier curves for clinical recurrence-free survival according to BED (198.3 Gy vs 150 Gy)

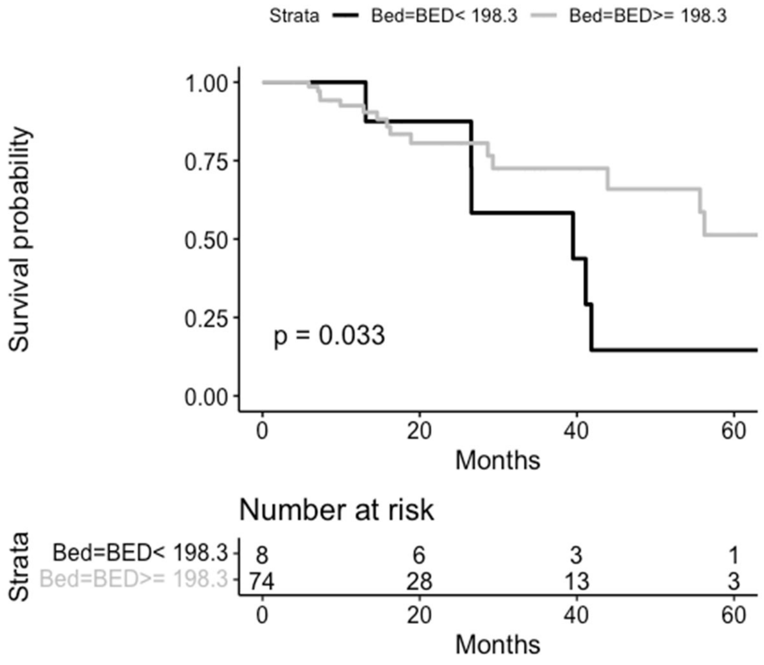

Figure S4. Kaplan-Meier curves for clinical recurrence-free survival according to GS

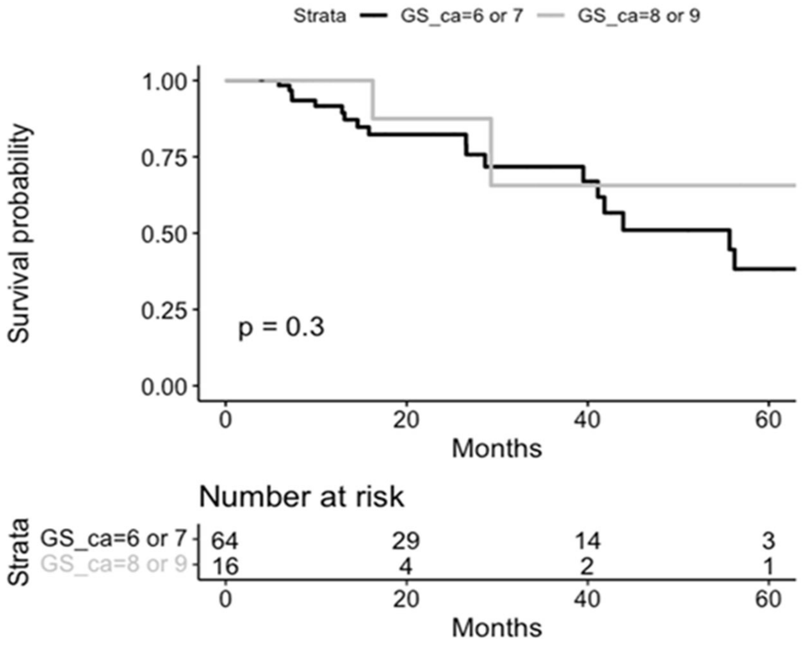

Figure S5. Kaplan-Meier curves for clinical recurrence-free survival according to concomitant ADT administration (ADT yes vs no)

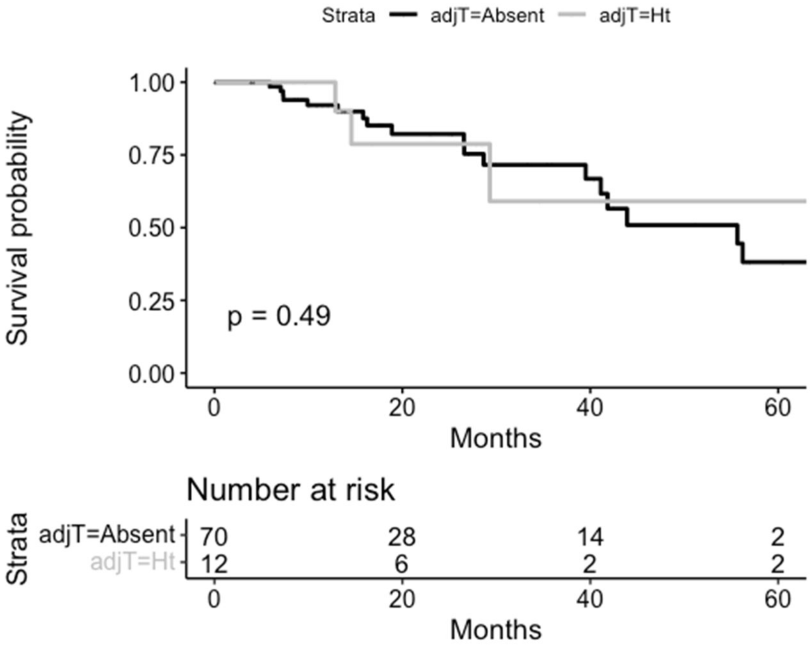

Supplement: Supplementary file 1 [file life-14-00870-s001.zip › life-3056108-supplementary.pdf]
